# Comprehensive Evaluation of Multi-Omics Clustering Algorithms for Cancer Molecular Subtyping

**DOI:** 10.3390/ijms26030963

**Published:** 2025-01-23

**Authors:** Juan Wang, Lingxiao Wang, Yi Liu, Xiao Li, Jie Ma, Mansheng Li, Yunping Zhu

**Affiliations:** 1 School of Basic Medical Sciences, Anhui Medical University, Hefei 230032, China; wwang_juan@163.com; 2 State Key Laboratory of Medical Proteomics, Beijing Proteome Research Center, National Center for Protein Sciences (Beijing), Beijing Institute of Lifeomics, Beijing 102206, China; wanglingxiao@ncpsb.org.cn (L.W.); leoicarus@163.com (Y.L.); lixiaobioinfo@163.com (X.L.); majie@ncpsb.org.cn (J.M.)

**Keywords:** multi-omics, cancer subtyping, clustering algorithm, accuracy-weighted average index

## Abstract

As a highly heterogeneous and complex disease, the identification of cancer’s molecular subtypes is crucial for accurate diagnosis and personalized treatment. The integration of multi-omics data enables a comprehensive interpretation of the molecular characteristics of cancer at various biological levels. In recent years, an increasing number of multi-omics clustering algorithms for cancer molecular subtyping have been proposed. However, the absence of a definitive gold standard makes it challenging to evaluate and compare these methods effectively. In this study, we developed a general framework for the comprehensive evaluation of multi-omics clustering algorithms and introduced an innovative metric, the accuracy-weighted average index, which simultaneously considers both clustering performance and clinical relevance. Using this framework, we performed a thorough evaluation and comparison of 11 state-of-the-art multi-omics clustering algorithms, including deep learning-based methods. By integrating the accuracy-weighted average index with computational efficiency, our analysis reveals that PIntMF demonstrates the best overall performance, making it a promising tool for molecular subtyping across a wide range of cancers.

## 1. Introduction

Cancer is a disease with complex etiology and high heterogeneity, necessitating its classification into distinct types or subtypes based on various characteristics. In recent years, with the widespread adoption of high-throughput technologies and the continuous increase in omics data, cancer subtyping has progressively shifted from a traditional focus on disease sites and histomorphological features to a more molecularly driven approach [1]. Initially, molecular subtyping of cancer primarily focused on single-omics data, which, while providing insights into molecular alterations at specific levels, struggled to offer a comprehensive understanding of the full spectrum of cancer biology. Multi-omics integration can uncover how various molecular entities and biological processes interact and interrelate [2]. In order to better investigate the complex regulatory mechanisms underlying cancer, researchers have increasingly turned to the integration of multi-omics data for molecular subtyping analysis. To date, numerous multi-omics clustering algorithms have been proposed for the molecular subtyping of cancer. These algorithms are typically classified into one or more of the following categories based on their underlying principles: similarity, fusion, matrix factorization, Bayesian, network, dimensionality reduction, deep learning, and multivariate [3,4,5].

The analysis of multi-omics data is prone to various sources of error, bias, and uncertainty that can affect their quality and validity, so methods for their analysis must be rigorously and transparently evaluated to ensure reliability and accuracy [2]. The evaluation and comparison of multi-omics clustering methods remains challenging due to the absence of a gold standard. Currently, some progress has been made in this area. For example, Tini et al. [6] tested several integration methods, MCCA, MCIA, MFA, JIVE, and SNF, for classifying patients with varying signal strengths and noise levels using both real datasets (mouse liver, platelet reactivity, and breast cancer datasets) and simulated datasets. They evaluated classification performance using F-scores and accuracy indices. Similarly, Chauvel et al. [7] evaluated the ability of algorithms such as iCluster, moCluster, iNMF, JIVE, MDI, and BCC to recover the correct number of clusters and simulated clusters at the public and data-specific levels using simulated datasets, with validation on a breast cancer dataset. However, most of the existing studies have not specifically focused on cancer multi-omics datasets, nor have they assessed the generalizability of evaluation metrics across a broad spectrum of cancers. The majority of research has primarily concentrated on traditional methods, with less emphasis on the application and evaluation of emerging multi-omics clustering algorithms, such as those based on deep learning. This highlights the need for the development of a robust evaluation framework that can be broadly applied to multi-omics data across various cancer types, representing a promising direction for future research.

**Figure 1:**
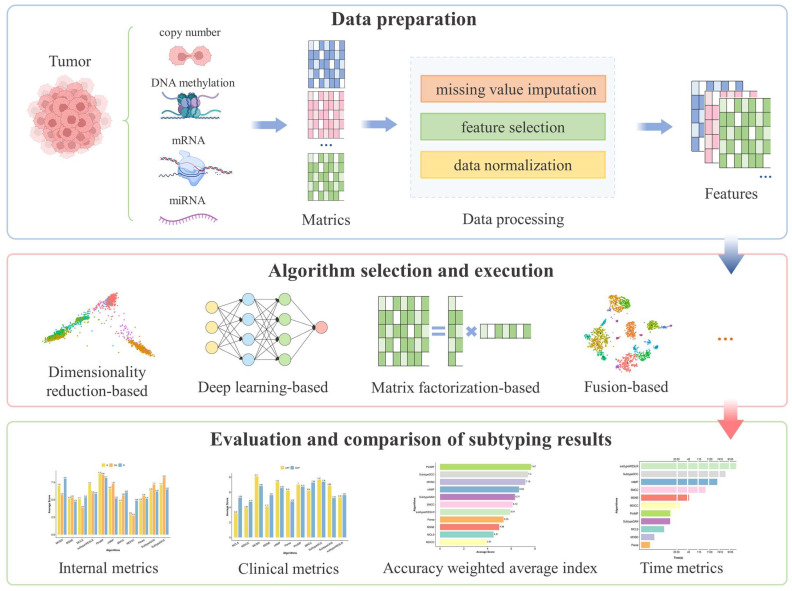
Comprehensive evaluation process of multi-omics clustering algorithms.

Under this situation, we developed a comprehensive evaluation process for multi-omics clustering algorithms and introduced a new metric, the accuracy-weighted average index (AWA). By integrating multi-omics data from a diverse set of cancers, we conducted a detailed comparative analysis of 11 state-of-the-art multi-omics clustering algorithms (MOSD [8], MSNE [9], MCLS [10], subtype-WESLR [11], PIntMF [12], nNMF [13], SMCC [14], MDICC [15], Parea [16], Subtype-GAN [17], and Subtype-DCC [18]), specifically focusing on their performance in the molecular subtyping of cancer (Figure 1). This evaluation not only helps identify the strengths and limitations of each algorithm but also provides valuable insights for guiding future algorithmic evaluation in this field.

## 2. Results

### 2.1. Internal Metrics of Clustering Algorithm

**Figure 2:**
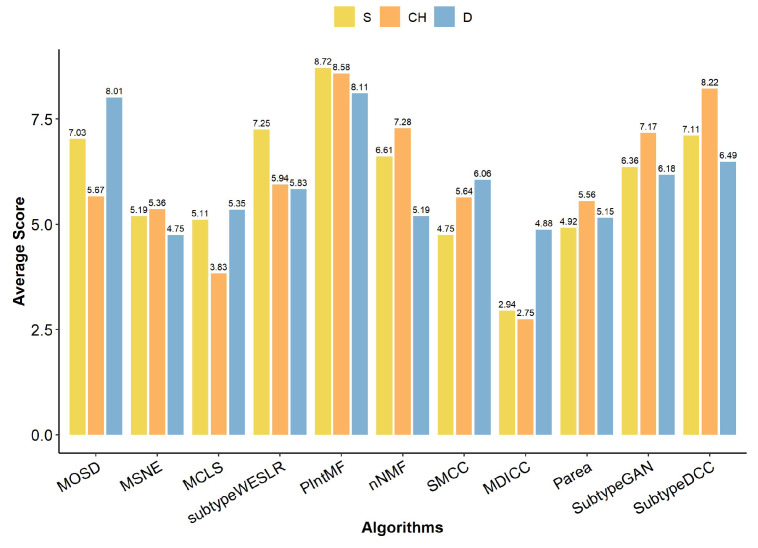
Internal metrics of clustering algorithm. The internal metrics (S, CH, D) are represented by bars of different colors. The height of each bar corresponds to the average score of each internal metric across the 9 cancer datasets.

Herein, we first calculated the internal metrics (silhouette coefficient (S), Calinski–Harabasz index (CH), and Dunn’s index (D) values) for 11 multi-omics clustering algorithms applied to each of the nine TCGA cancer datasets (BRCA, BLCA, KIRC, LUAD, PAAD, SKCM, STAD, UCEC, and UVM). We then evaluated the clustering effectiveness of the algorithms by averaging the internal metric scores across the cancer datasets (Figure 2). The results showed that PIntMF achieved the highest average scores for internal metrics, indicating that its clustering performance was superior across the cancer datasets compared to the other 10 algorithms. Additionally, Subtype-DCC ranked in the top three for all three internal evaluation metrics, demonstrating similarly strong clustering performance. In contrast, MDICC and MCLS received relatively low scores, suggesting poorer clustering performance.

### 2.2. Clinical Metrics of Clustering Algorithm

**Figure 3:**
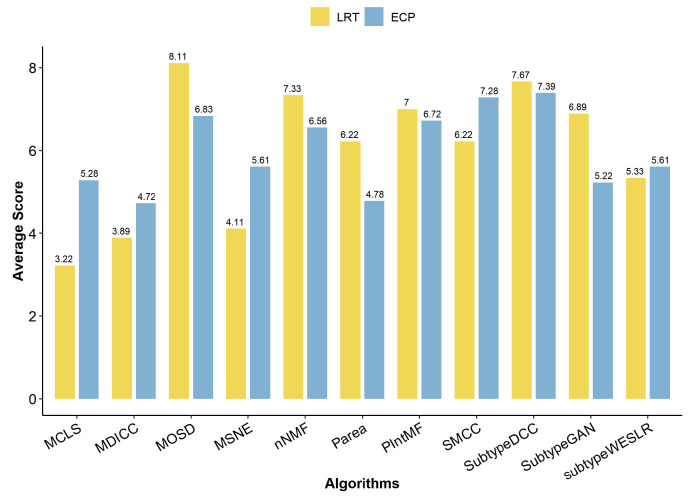
Clinical metrics of clustering algorithm. The clinical metrics (LRT and ECP) are represented by bars of different colors. The height of each bar indicates the average score for each clinical metric across the 9 cancer datasets.

Clinical performance between molecular subtypes is primarily assessed by survival differences and clinical label enrichment, quantified by the log rank test (LRT) and enriched clinical parameters (ECP) values, respectively. To minimize bias in the enrichment analysis, we selected a consistent set of clinical information across all cancer datasets, including gender, age at diagnosis, pathologic T, pathologic M, pathologic N, and pathologic stage. The clinical metric scores reveal that SubtypeDCC achieved the highest scores for both LRT and ECP (Figure 3), indicating its superior ability to identify subtypes with clinically significant differences. Similarly, MOSD performed well, particularly excelling in the ECP metric, where it outperformed all other algorithms. In contrast, MDICC had lower clinical metric scores, suggesting it identifies subtypes with less pronounced clinical differences.

### 2.3. The Accuracy-Weighted Average Index of Clustering Algorithm

**Figure 4:**
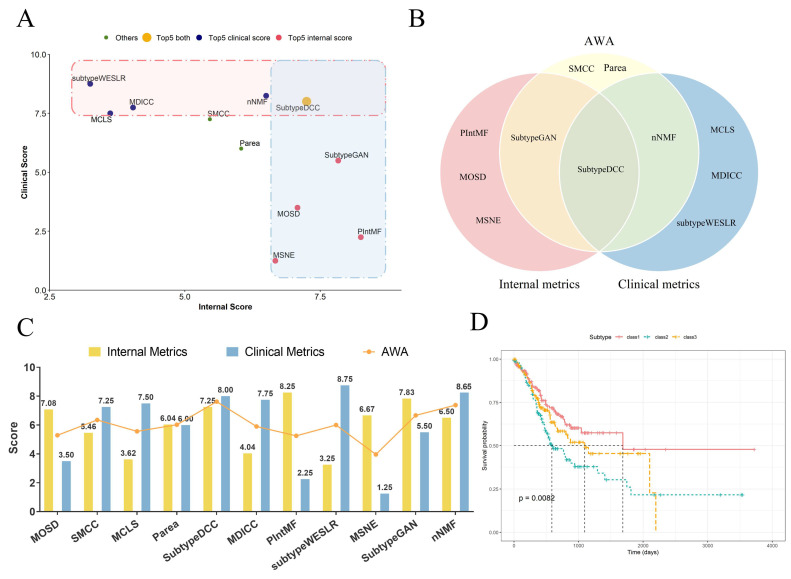
Metrics of clustering algorithms on the STAD dataset: (**A**) Plot showing the metrics of clustering algorithms on the STAD dataset, with the horizontal axis representing internal metrics and the vertical axis representing clinical metrics. The top five algorithms, ranked by internal or clinical metrics, are highlighted with color blocks. (**B**) Venn diagram illustrating the overlap of the top five algorithms selected based on internal metrics, clinical metrics, and AWA scores for the STAD dataset. (**C**) Bar chart displaying the internal metrics, clinical metrics, and AWA scores for all 11 algorithms on the STAD dataset. The yellow and blue bars represent the average internal metrics and clinical metrics for each algorithm, respectively, with the corresponding values labeled at the top of the bars. The endpoints of the line indicate the average AWA scores for the algorithms. The horizontal axis corresponds to the 11 algorithms, while the vertical axis represents the scores. (**D**) Kaplan–Meier survival plot for the clustering result of SubtypeDCC on the STAD dataset. The black dashed lines represent the survival times corresponding to a 50% cumulative survival probability for each of the three subtypes.

Although optimal algorithms can be found based on clustering effects and clinical performance through internal and clinical metrics, respectively, discrepancies remain when considering the overall perspective. We ranked the algorithms based on their internal and clinical scores separately across different cancer datasets and selected the top five scorers in each category. Interestingly, algorithms selected based on internal metrics (internal algorithms) did not align with those selected based on clinical metrics (clinical algorithms) (Figure 4A). This observation suggests that such screening methods might highlight algorithms that excel in one aspect (either clustering effect or clinical performance) but perform poorly in the other. Moreover, these methods could miss high-quality algorithms that maintain balanced performance across both aspects. For example, in the STAD dataset, MOSD and MSNE ranked in the top five for internal scores (7.08 and 6.67, respectively), but their clinical scores (3.5 and 1.25) placed them at the bottom (Figure 4A).

To address this limitation, we propose the AWA value, which assigns different weights to internal and clinical metrics (I/C) and performs weighted averaging to synthesize the clustering effect and clinical performance of the algorithm. With the set weights (I/C = 50%/50%) to calculate the AWA, we identify SubtypeDCC, nNMF, SubtypeGAN, SMCC, and Parea as the top performers in both clustering effect and clinical performance for the STAD dataset (Figure 4B,C). Notably, while SMCC and Parea were not screened out on the internal or clinical scores, their balanced performance in both aspects outperformed over half of their counterparts. Additionally, SubtypeDCC is the preferred algorithm for clustering the STAD dataset, as it effectively combines both clustering effectiveness and clinical performance. We performed the survival analyses of clustering results on the STAD dataset from PIntMF (top-ranked for internal metrics), subtypeWESLR (top-ranked for clinical metrics), and SubtypeDCC (top-ranked for AWA scores). The clustering result derived from SubtypeDCC shows the highest confidence level with survival analyses (SubtypeDCC, *p* = 0.0082 (Figure 4D); subtypeWESLR, *p* = 0.038 (Figure S5); PIntMF, *p* = 0.66), suggesting that AWA can balance the difference between internal and clinical indicators and provide a more effective measurement of the disease-relative characteristics.

**Figure 5:**
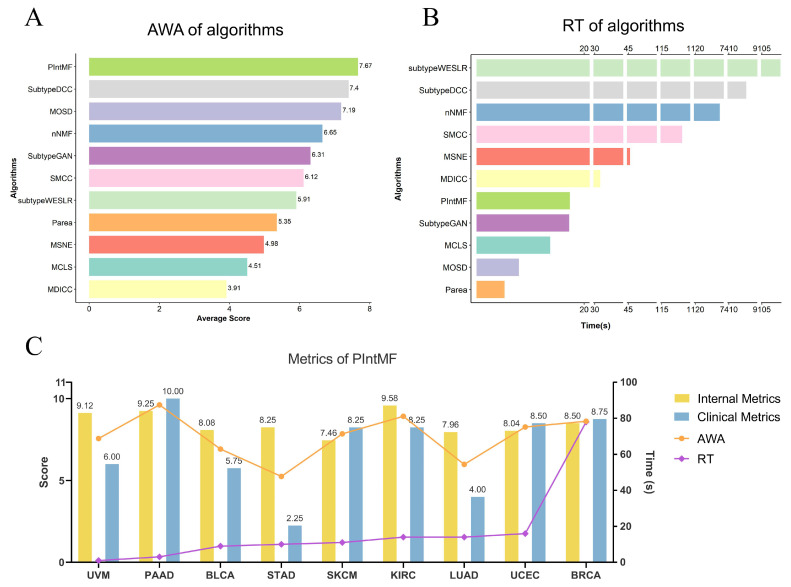
Average AWA score and running time (RT) for 11 clustering algorithms on 9 cancer datasets: (**A**) Bar diagram showing the average AWA scores for 11 clustering algorithms on 9 cancer datasets, sorted by score from highest to lowest. (**B**) Bar diagram illustrating the average running time for the 11 clustering algorithms on the 9 cancer datasets, sorted by runtime from longest to shortest. (**C**) Overview of the internal metrics, clinical metrics, AWA score, and runtime for PIntMF across the 9 cancer datasets. The yellow and blue bars represent the average internal metrics and clinical metrics for each algorithm, respectively, with the corresponding values labeled at the top of the bars. The endpoints of the line indicate the average AWA scores (denoted by dot endpoints on the orange line) and runtime (denoted by diamond endpoints on the purple line) for the algorithms. The horizontal axis represents the 11 algorithms, the left vertical axis displays the internal metrics, clinical metrics, and AWA scores, and the right vertical axis shows the run time.

Meanwhile, we calculated and ranked the AWA scores for 11 algorithms applied to nine different cancer datasets (Figure 5A), with the complete set of AWA scores provided in Figure S1. Although the accuracy of each algorithm varies significantly across different cancer datasets, PIntMF, which achieves the highest AWA score, performs superiorly across most datasets, particularly excelling in the PAAD, UCEC, and BRCA datasets (with AWA scores of 9.62, 8.92, and 8.62, respectively). However, PIntMF performed relatively poorly in the STAD and LUAD datasets (with AWA scores of 5.25 and 5.98), primarily due to its low clinical scores in these datasets (2.25 and 4.00, respectively). In contrast, SubtypeDCC, which has the second-highest AWA score, performs consistently across all datasets (with AWA scores above 6.5 in all cases) and outperforms PIntMF in the BLCA, STAD, SKCM, and LUAD datasets (Figure S1), yet it is less efficient in terms of runtime. In addition, the lower-ranked algorithms MDICC and MCLS scored below 5 in most datasets, particularly ranking among the worst performers in the BLCA, KIRC, and BRCA datasets. To verify the reliability of the ranking, we calculated the AWA scores under various weight settings (Figure S2). Notably, PIntMF and SubtypeDCC consistently maintained their leading performance across different weights.

In addition to achieving high accuracy, researchers highly value multi-omics clustering algorithms that are efficient and require less computation time. Here, we calculated the average runtime of the 11 algorithms across nine cancer datasets. As shown in Figure 5B, with the exception of Subtype-WESLR, Subtype-DCC, nNMF, and SMCC, the average runtime of the remaining seven algorithms is under 50 s, indicating their high operational efficiency.

### 2.4. Molecular Subtyping Performance of PIntMF

**Figure 6:**
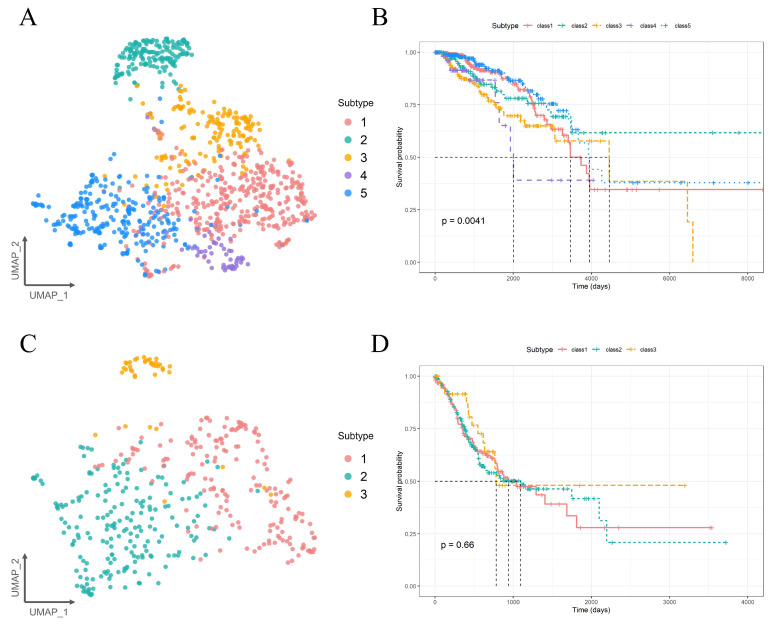
Clustering performance and clinical performance of PIntMF: (**A**) UMAP visualization of latent variables generated by PIntMF based on the BRCA dataset. (**B**) Kaplan–Meier survival plot for the clustering result of PIntMF on the BRCA dataset. The black dashed lines represent the survival times corresponding to a 50% cumulative survival probability for each of the five subtypes. (**C**) UMAP visualization of latent variables generated by PIntMF based on the STAD dataset. (**D**) Kaplan–Meier survival plot for the clustering result of PIntMF on the STAD dataset. The black dashed lines represent the survival times corresponding to a 50% cumulative survival probability for each of the three subtypes.

To assess whether the AWA scores accurately reflect both the clustering effect and clinical performance of the algorithm, we conducted UMAP dimensionality reduction and survival analysis using the PIntMF algorithm on the BRCA dataset (AWA score: 8.62, indicating high accuracy) and the STAD dataset (AWA score: 5.25, indicating low accuracy). As shown in Figure 6, the BRCA dataset was successfully divided into five distinct clusters, with significant survival differences between these clusters (*p* = 0.0041), among which Cluster 2 exhibited the best prognostic effect. In contrast, the STAD dataset was divided into three clusters, but its UMAP visualization revealed obvious cross-mixing and the survival differences between the clusters were not significant (*p* = 0.66). This suggests that PIntMF performs poorly for subtyping STAD and may not be effective in supporting prognosis for this subtype. These findings are consistent with the observed AWA scores. Therefore, the AWA score serves as a reliable metric for evaluating the performance of the algorithm, facilitating both cancer subtyping and its associated prognosis.

## 3. Discussion

In recent years, the field of Artificial Intelligence (AI) has transitioned from primarily theoretical research to real-world applications. [19]. For example, the FDA approved automated detection, counting, and computer-generated analysis of the HER2 gene for therapeutic determination in breast cancer [20]. Esteva et al. trained a CNN dataset to recognize nonmelanoma skin cancer versus benign seborrheic keratosis and melanoma versus benign nevi. They project their results to a practical application of incorporating deep neural networks into clinician’s mobile devices to yield diagnoses beyond the confines of the office/clinic [21]. However, the ability of AI to effectively assist in clinical decision-making depends on the performance of prediction, recognition, or classification models.

Molecular subtyping of cancer plays a crucial role in enabling precise diagnosis and personalized treatment for patients, which also requires high precision in subtyping algorithms to support decision-making. With the rapid advancement of multi-omics clustering algorithms, the need for effective evaluation and comparison of these methods has become increasingly important. In this study, we established a comprehensive framework for evaluating multi-omics clustering algorithms. We assessed the clustering performance and clinical relevance of 11 algorithms across nine TCGA cancer datasets using both internal and clinical metrics. During the evaluation, we observed that while some algorithms had very similar performance across these two types of metrics, many others exhibited significant discrepancies between their internal and clinical scores. This variation posed challenges in evaluating and selecting the best-performing algorithm, making it difficult to quickly identify the algorithm with the highest accuracy.

Therefore, we propose a new evaluation metric, AWA, which measures the accuracy of multi-omics clustering algorithms by considering both their clustering effectiveness and the clinical performance of the resulting molecular subtypes. This metric enables a comprehensive assessment of algorithm performance in cancer molecular subtyping. Additionally, the optimal clustering algorithm varies across different cancer datasets. Among the nine cancer datasets analyzed in this study, PIntMF achieved the highest AWA score, demonstrating strong overall performance, particularly in the PAAD, UCEC, and BRCA datasets, where it can be recommended as the first-choice clustering algorithm (Figure 5C). Furthermore, the subtyping performance of PIntMF on the BRCA and STAD datasets further demonstrates that AWA serves as a reliable metric for evaluating the algorithm’s subtyping performance. Further, to validate the reliability and generalizability of the comprehensive evaluation process, we applied our analysis to two non-TCGA datasets: PAT and HCC datasets. The results show that our strategy can achieve an accurate evaluation of the performance of multi-omics clustering algorithms on the supplemental datasets. PIntMF, the best clustering algorithm identified by the evaluation process in TCGA datasets, consistently performs well in the non-TCGA datasets containing additional omics data (Figures S3 and S4).

By combining the AWA scores with the operational efficiency of each algorithm, we found that PIntMF delivers the best overall performance (Figure 5). This performance may be attributed to the design idea of PIntMF. In terms of clustering performance, a key advantage of this method is its ability to automatically tune the lasso penalties for both variable and sample matrices. For operational efficiency, PIntMF utilizes the faster GLMNet framework to infer the matrix *H^k^* and optimizes algorithm initialization using the SNF algorithm [12]. These features enable PIntMF to effectively address the distributional heterogeneity of multi-omics data, with its performance being particularly exceptional in handling the PAAD and BRCA datasets. In contrast, other algorithms showed certain limitations: SubtypeDCC and MOSD exhibited high accuracy but poor running efficiency, while MDICC had lower accuracy but better operational efficiency. However, each algorithm has its limitations in application. For instance, when applied to large datasets, the operational efficiency of PIntMF decreases significantly [12]; other algorithms that exhibit lower accuracy but better runtime efficiency, such as MDICC, may offer more effective support for disease subtype clustering.

Overall, we construct a comprehensive evaluation process to identify ideal algorithms for cancer molecular subtyping and provide a theoretical foundation for the development of computer-aided tools to support cancer diagnosis and treatment. After being evaluated by multiple datasets, PIntMF is identified as a highly accurate and operationally efficient cancer subtyping algorithm that can be integrated into computer-aided tools for analyzing patient-derived multi-omics data, assisting in the diagnosis and treatment of specific cancers, such as PAAD, BRCA, and UCEC.

Notably, in this study, the clustering effect of the algorithm was considered to be of equal importance to the clinical performance. To calculate the overall evaluation, we assigned equal weights to the internal and clinical metrics. However, this may not represent the optimal weighting for each cancer dataset, and these parameters could be adjusted in future studies depending on the specific cancer datasets or research focus. In addition, the K-values for the cancer datasets were selected based on existing studies; we recognize that the performance of clustering algorithms could be further improved by optimization of K-values on different datasets. Additionally, the current evaluation process mainly focused on omics data of copy number, DNA methylation, mRNA, and miRNA data for nine cancers, which may lead to limitations in the application of the evaluation process to other types of omics data and diseases. In future studies, we will try to include a broader range of multi-omics data (e.g., proteomics, single-cell omics, etc.) and validate and improve the evaluation process across a wider array of diseases. The characteristics of clustering algorithms, such as interpretability and the issues related to deep learning models, including overfitting and underfitting results, that were not included in the current study, should also be considered.

## 4. Materials and Methods

In this study, a comprehensive evaluation process of multi-omics clustering algorithms was constructed (Figure 1). The process began with the preparation and preprocessing of the omics data, followed by the selection and execution of clustering algorithms. Finally, the molecular subtyping performance of the different algorithms was thoroughly assessed based on the AWA and running efficiency.

### 4.1. Data Sources and Preprocessing

The Cancer Genome Atlas (TCGA), a cancer research project created in 2006 by the National Cancer Institute (NCI) and the National Human Genome Research Institute (NHGIR), contains genomic, transcriptomic, epigenomic, and proteomic data across 33 different cancer types [22]. In the current study, we utilized 9 TCGA cancer datasets, as provided by Yang et al. [17]. These datasets collectively include 4027 tumor samples from nine cancer types: breast invasive carcinoma (BRCA, 1031 samples), bladder urothelial carcinoma (BLCA, 399 samples), kidney renal clear cell carcinoma (KIRC, 488 samples), lung adenocarcinoma (LUAD, 490 samples), pancreatic adenocarcinoma (PAAD, 176 samples), skin cutaneous melanoma (SKCM, 446 samples), stomach adenocarcinoma (STAD, 407 samples), uterine corpus endometrial carcinoma (UCEC, 510 samples), and uveal melanoma (UVM, 80 samples). Each dataset includes four types of omics data for all samples: copy number, DNA methylation, mRNA, and miRNA. The large sample size and comprehensive clinical information in these datasets ensure the stability and reliability of the subsequent molecular subtyping analysis. To minimize bias in the clinical analysis, we selected a consistent set of clinical information across all cancer datasets, including gender, age at diagnosis, pathologic T, pathologic M, pathologic N, and pathologic stage.

After data collection, we performed preprocessing on the four omics data types: (1) Copy number data: removing duplicate regions from the original data and constructing features based on the correspondence between samples and genomic regions. (2) DNA methylation data: combining features with β values ≥ 0.3 from DNA methylation arrays HM27 and HM450. (3) mRNA and miRNA data: after log2 transformation, poorly expressed genes were removed using median absolute deviation and performing feature dimensionality reduction through variance threshold. Moreover, missing values were imputed using the sample mean of each omics data, and data were normalized by removing the mean of the features and scaling them to unit variance. After preprocessing, we obtained a total of 3105 copy number features, 3139 DNA methylation features, 3217 mRNA features, and 383 miRNA features.

To further validate the reliability and generalizability of the comprehensive evaluation process, we supplemented our analysis with two non-TCGA datasets: the pediatric brain tumor (PBT) dataset (214 samples) from the UCSC Xena browser (https://xenabrowser.net/ (accessed on 20 September 2024)) and the hepatocellular carcinoma (HCC) dataset (159 samples) from the National Omics Data Encyclopedia (NODE, https://www.biosino.org/node/ (accessed on 25 September 2024)). The PBT dataset comprises three omics types: transcriptomics, proteomics, and phosphoproteomics, while the HCC dataset includes four omics types: genomics (copy number), transcriptomics, proteomics, and phosphoproteomics. Preprocessing methods for copy number and transcriptomics data followed the procedures described above. For proteomics and phosphoproteomics data, features with more than 50% missing values were excluded, and the remaining missing values were imputed using the mean. Feature selection and normalization were conducted using the median absolute deviation (MAD) and median normalization methods, respectively.

It is important to note that multi-omics clustering algorithms for molecular subtyping of cancers typically use unsupervised clustering methods. To ensure the fairness and consistency of the evaluation results, the number of clusters (K-value) for each cancer dataset was set based on the reasonable number of subtypes identified in previous studies (Table 1).

### 4.2. Multi-Omics Clustering Algorithm Selection

In recent years, researchers have developed a wide range of multi-omics clustering algorithms, each employing different strategies. Even when applied to the same cancer dataset, these algorithms often yield inconsistent subtyping results. As a result, evaluating and comparing different algorithms becomes crucial for ensuring reliable and accurate subtyping.

This study evaluated the performance of 11 multi-omics clustering algorithms for comprehensive cancer molecular subtyping. The selection was based on the multi-omics clustering algorithms published in the last five years and the availability of implementable code. A brief description of each of the 11 algorithms is provided below:

1. MOSD [8] first creates affinities for each data type using local scaling. The affinities are then linearly combined into a network by assigning weights to each omics. Finally, spectral clustering is applied to the self-diffusion-enhanced similarity network to identify cancer subtypes.
2. MSNE [9] first constructs similarity networks of samples for complete or partial multi-omics data. Then, the integrated similarity of samples is captured through random walk on multiple similarity networks. Finally, the cancer subtypes are obtained with the Skip-gram method by projecting the samples into a low-dimensional space for k-mean clustering.
3. MCLS [10] first utilizes complete multi-omics data to construct a latent subspace using principal component analysis (PCA)-based feature extraction and singular value decomposition (SVD). Then, a projection matrix of each omics is learned to project the incomplete multi-omics data to the latent subspace. Finally, the samples are clustered using spectral clustering in the latent subspace.
4. Subtype-WESLR [11] is based on a sparse subspace learning framework. First, it employs a weighted ensemble strategy to fuse base clustering obtained from different methods as prior knowledge. Then, the sample feature profiles of each data type are projected to a common latent subspace corresponding to the subspace consistency. Finally, the common subspace is optimized by an iterative method to identify cancer subtypes.
5. PIntMF [12] is a matrix factorization model with non-negativity and sparsity constraints. First, the original matrix is decomposed into a product of a common basis matrix (*W*) and a specific coefficient matrix (*H^k^*). Then, sparsity is added to *W* and Hk via lasso penalization and equality constraints are applied to improve interpretability. Finally, *W* and *H^k^* are iteratively updated until the similarity of *W* is stable, and hierarchical clustering is performed on *W* to obtain the sample subtypes.
6. nNMF [13] combines the strengths of intNMF [34] and SNF [35]. It first uses intNMF to construct a stable consensus matrix for each data type. Then, theses consensus matrices are integrated into a single consensus matrix by SNF. Finally, spectral clustering is performed on this single consensus matrix to determine cancer subtypes.
7. SMCC [14] first constructs sample-sample similarity networks based on Euclidean distance. Then, it integrates weighted least squares, low-rank subspace representation, and entropy to fuse networks. Finally, co-regularization is used to measure and minimize the distribution difference between the similarity networks and the fused network, and cancer subtypes are obtained through clustering.
8. MDICC [15] first constructs affinity matrices for different omics data based on Gaussian kernel functions. After fusing them using low-rank subspace representation and entropy, the integrated matrix is clustered using K-means++ to obtain cancer subtypes.
9. Parea [16] is based on multi-view hierarchical clustering and data fusion. It first selects hierarchical clustering methods to represent each omics data into separate views. Then, hierarchical clustering is used again to identify cancer subtypes by creating a fusion object.
10. Subtype-GAN [17] is a deep adversarial learning method based on a multi-input multi-output neural network. First, the features of each omics data are extracted from relatively independent layers. Then, after inputting these features into the same shared layer, the subtypes of the samples are obtained by consensus GMM clustering through the hidden factors of the shared layer.
11. Subtype-DCC [18] combines deep clustering and decoupled comparison learning. First, data pairs are constructed from the pair construction backbone (PCB) through data augmentation, and features are extracted from the data augmentation using a shared deep neural network. Then, the instance-level contrastive head (ICH) and the cluster-level contrastive head (CCH) are used for contrastive learning in the row and column spaces of the feature matrix, respectively. After training, the CCH is used to predict cancer subtypes.

As shown in Table 2, the operating principles of each multi-omics clustering algorithm are different. Based on the classification framework proposed by Subramanian and Rappoport et al. [3,4], we categorized the 11 algorithms into five main groups: similarity-based clustering methods, dimensionality reduction-based clustering methods, matrix factorization-based clustering methods, fusion-based methods, and deep learning-based clustering methods.

### 4.3. Metrics for Evaluating Subtyping Performance

Currently, commonly used metrics for evaluating cancer subtyping algorithms include clustering internal metrics or clinical metrics and, in some cases, runtime to assess the algorithm’s operational performance. Internal metrics include the silhouette coefficient (S) [36], the Calinski–Harabasz index (CH) [37], and the Dunn’s index (D) [38]. S means the pairwise differences in inter- and intra-cluster distances, CH represents the average of inter- and intra-cluster sums of squares, and D is the ratio of the minimum pairwise distance between clusters to the maximum diameter within a cluster. All the internal metrics do not rely on any external information and only measure the performance of clustering through intra-cluster compactness and inter-cluster separation. Clinical metrics include survival differences between subtypes obtained by the log rank test (LRT) [39] and the number of enriched clinical parameters (ECP) obtained by the χ^2^ test [40] and the Kruskal–Wallis test [41]. The formulas for all clustering internal metrics or clinical metrics used in this study are provided in Table 3.

When comparing the subtyping performance of algorithms, the clinical significance of inter-subtyping is as important as the clustering effect itself. To integrate both the clustering performance of the algorithm and the clinical characteristics between subtypes, we propose a new evaluation metric, the accuracy-weighted average index (AWA). The AWA is defined as follows:

(1) $$AWA=\frac{\left(S_{S}+S_{CH}+S_{D}\right)\times w_{1}+\left(S_{LRT}+S_{ECP}\right)\times w_{2}}{3w_{1}+2w_{2}}$$

where *S_S_*, *S_CH_*, and *S_D_* represent S, CH, and D of the internal metrics, respectively. *S_LRT_* and *S_ECP_* mean LRT and ECP in the clinical metrics. Moreover, *w_1_* and *w_2_* are weights of the internal and clinical metrics, respectively.

## 5. Conclusions

In this study, we developed a comprehensive framework for evaluating multi-omics clustering algorithms and introduced a novel metric for subtyping performance, the accuracy-weighted average index, which assigns different weights to clustering effectiveness and clinical performance. Furthermore, we comprehensively compared the molecular subtyping performance of 11 frontier multi-omics clustering algorithms on nine TCGA cancer datasets. Our findings indicate that PIntMF is the preferred algorithm for molecular typing in most cancers owing to its superior clustering accuracy and operational efficiency.

## Figures and Tables

**Table 1:** K value of 11 cancer datasets.

Datasets	K Value	References
BRCA	5	[23]
BLCA	5	[24]
KIRC	4	[25]
LUAD	3	[26]
PAAD	2	[27]
SKCM	4	[28]
STAD	3	[29]
UCEC	4	[30]
UVM	4	[31]
PBT	8	[32]
HCC	3	[33]

**Table 2:** Classification and information of 11 multi-omics clustering algorithms.

Categories	Algorithms	Year	Implementation	Reference
Similarity-based clustering methods	MOSD	2024	https://github.com/DXCODEE/MOSD (accessed on 4 March 2024)	[8]
	MSNE	2021	https://github.com/GaoLabXDU/MSNE (accessed on 5 March 2024)	[9]
Dimensionality reduction-based clustering methods	MCLS	2023	https://github.com/ShangCS/MCLS (accessed on 9 March 2024)	[10]
	subtype-WESLR	2022	https://github.com/songwenjing123/subtype-WESLR (accessed on 2 April 2024)	[11]
Matrix factorization-based clustering methods	PIntMF	2022	https://github.com/mpierrejean/pintmf (accessed on 5 April 2024)	[12]
	nNMF	2020	R v4.3.2	[13]
Fusion-based clustering methods	SMCC	2024	https://github.com/yushanqiu/SMCC (accessed on 5 April 2024)	[14]
	MDICC	2022	https://github.com/yushanqiu/MDICC (accessed on 5 April 2024)	[15]
	Parea	2023	https://github.com/mdbloice/Pyrea (accessed on 5 April 2024)	[16]
Deep learning-based clustering methods	Subtype-GAN	2021	https://github.com/haiyang1986/Subtype-GAN (accessed on 6 April 2024)	[17]
	Subtype-DCC	2023	https://github.com/zhaojingo/Subtype-DCC (accessed on 6 April 2024)	[18]

**Table 3:** Metrics of subtyping performance evaluation.

Categories	Metrics	Formulas	Optimum
Internal metrics	S	$S=\frac{b-a}{max(a,b)}$ *a*: mean distance from the sample point to other points within the same cluster *b*: mean distance from the sample point to the nearest other cluster	Maximum
	CH	$CH=\frac{tr(B_{k})\times (n-k)}{tr(W_{k})\times (k-1)}$ *B**_k_***: inter-cluster covariance matrix *W_k_*: intra-cluster covariance matrix *n*: sample size *k*: number of clusters	Maximum
	D	$D=\frac{{min}_{i\neq j}(d\left(C_{i},C_{j}\right))}{{max}_{k}(diam(C_{k}))}$ *d*(*C_i_*, *C_j_*): inter-cluster distance *diam*(*C_k_*): cluster diameter	Maximum
Clinical metrics	LRT	Using log rank test to calculate the *p*-value	Minimum
	ECP	χ^2^ test discrete parameter enrichment Kruskal–Wallis test numerical parameter enrichment	Maximum
Time metrics	RT	Average running time	Minimum

## Data Availability

The original contributions presented in this study are included in the article/Supplementary Materials.

## Supplementary Materials

The following supporting information can be downloaded at: https://www.mdpi.com/article/10.3390/ijms26030963/s1.
