# Supplementary material for: Comprehensive Evaluation of Multi-Omics Clustering Algorithms for Cancer Molecular Subtyping
Source: Int J Mol Sci. 2025 Jan 23;26(3):963. doi: 10.3390/ijms26030963 (PMC11816650; doi:10.3390/ijms26030963)

Figure S1. AWA scores of all clustering algorithms. The rows of the heatmap represent cancer datasets, while the columns correspond to the algorithms. Stronger colors indicate higher AWA scores, with detailed scores labeled in each cell.

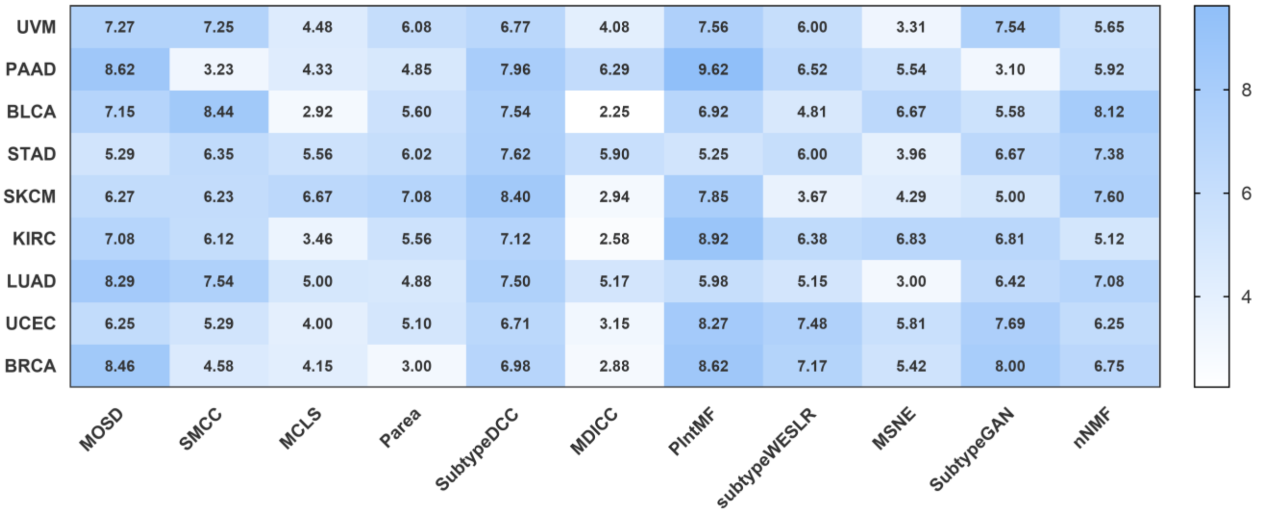

Figure S2. AWA scores of 11 clustering algorithms with different weights. The AWA scores of the 11 clustering algorithms in the bar chart are sorted from highest to lowest. The weights of the internal and clinical metrics are labeled at the top of each graph (e.g. I/C = 20%/80% represents a weighting of 20% for internal indicators and 80% for clinical indicators). Based on different weight values, the specific *w1* and *w2* are calculated (A: *w1* = 0.07, *w2* = 0.40; B: *w1* = 0.27, *w2* = 0.10; C: *w1* = 0.10, *w2* = 0.45; D: *w1* = 0.23, *w2* = 0.15; E: *w1* = 0.13, *w2* = 0.30; F: *w1* = 0.20, *w2* = 0.20).

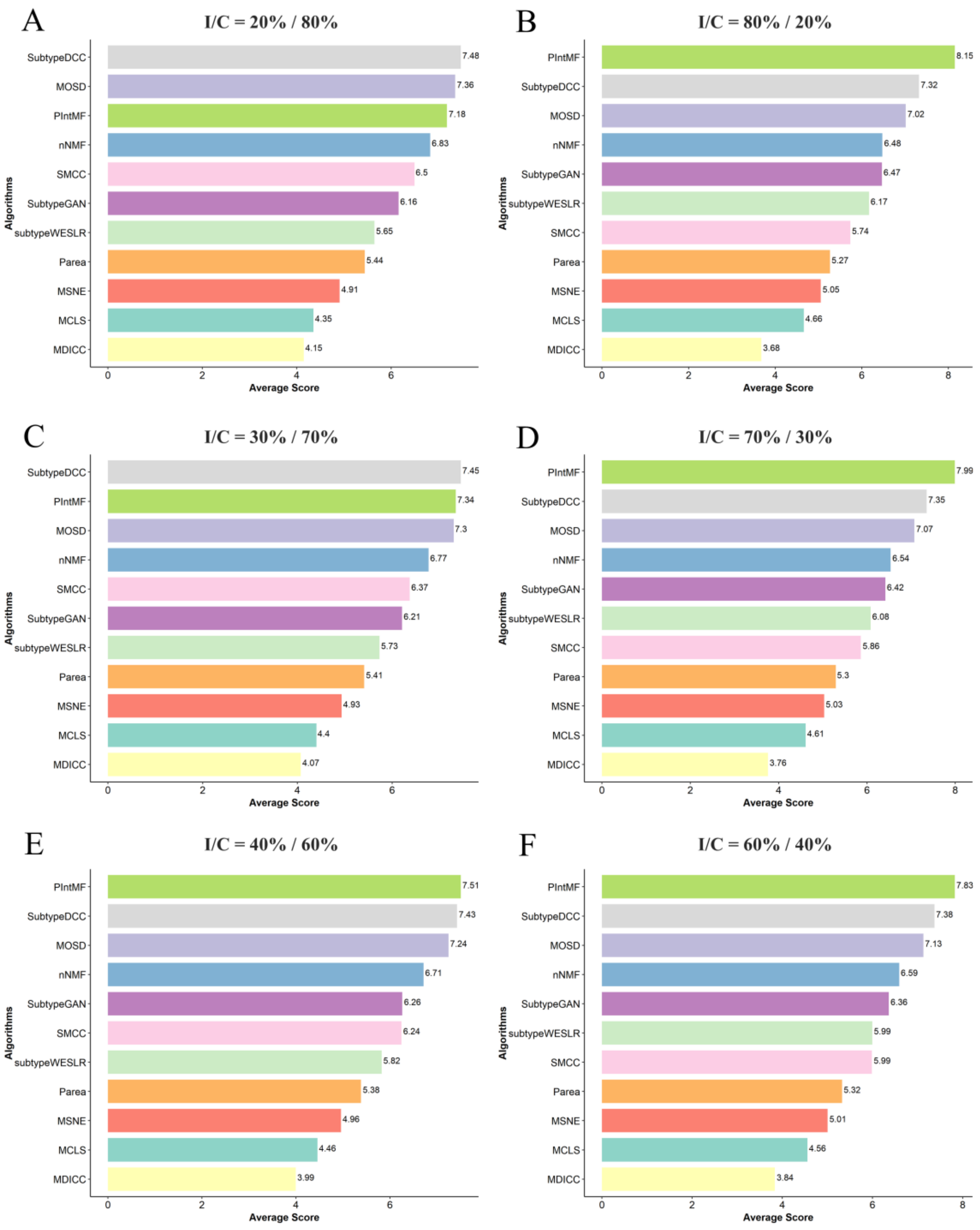

Figure S3. The internal metrics, clinical metrics, and AWA scores for all 11 algorithms on the HCC dataset. The yellow and blue bars represent the average internal metrics and clinical metrics for each algorithm, respectively, with the corresponding values labeled at the top of the bars. The endpoints of the line indicate the average AWA scores for the algorithms. The horizontal axis corresponds to the 11 algorithms, while the vertical axis represents the scores.

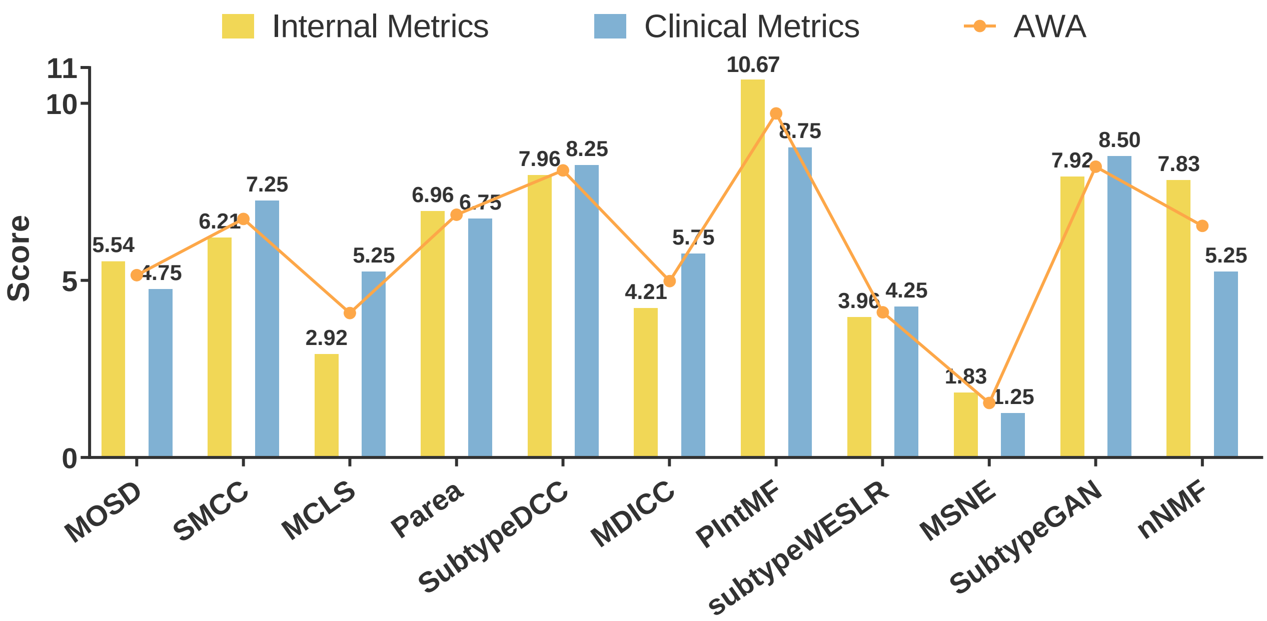

Figure S4. The internal metrics, clinical metrics, and AWA scores for all 11 algorithms on the PBT dataset. The yellow and blue bars represent the average internal metrics and clinical metrics for each algorithm, respectively, with the corresponding values labeled at the top of the bars. The endpoints of the line indicate the average AWA scores for the algorithms. The horizontal axis corresponds to the 11 algorithms, while the vertical axis represents the scores.

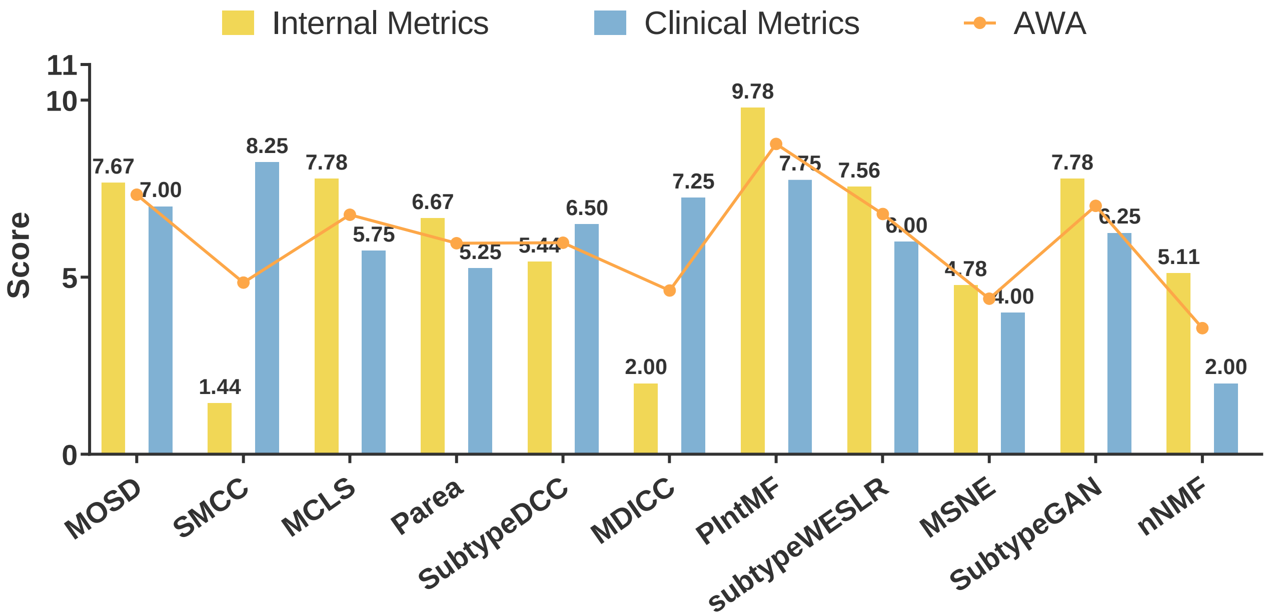

Figure S5. Kaplan-Meier survival plot for the clustering result of subtypeWESLR on the STAD dataset.

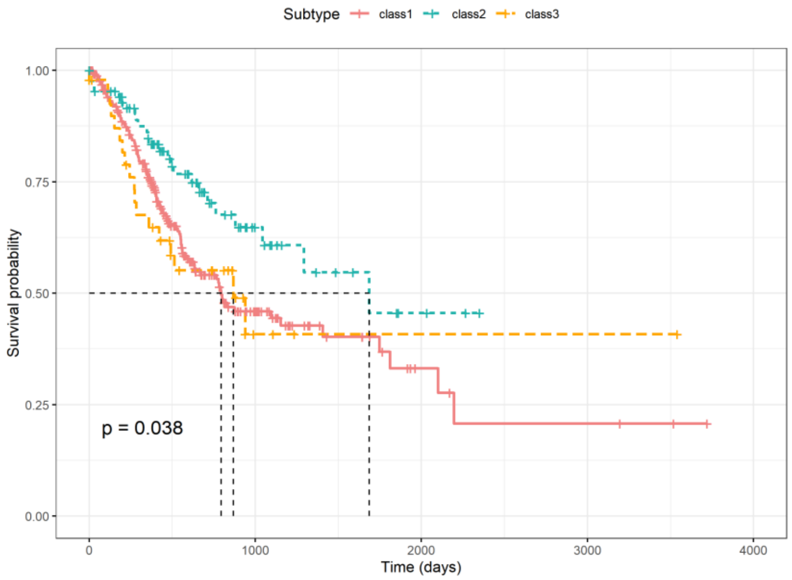

Supplement: Supplementary file 1 [file ijms-26-00963-s001.zip › ijms-3399310-supplementary.pdf]
